# Synergistic effect of *Dermatophagoides pteronyssinus* allergen and *Escherichia coli* lipopolysaccharide on human blood cells

**DOI:** 10.1371/journal.pone.0207311

**Published:** 2018-11-09

**Authors:** Yaroslav V. Radzyukevich, Ninel I. Kosyakova, Isabella R. Prokhorenko

**Affiliations:** 1 Laboratory of Molecular Biomedicine, Institute of Basic Biological Problems RAS, Pushchino, Russia Federation; 2 Hospital of Pushchino Scientific Center, Russian Academy of Sciences, Pushchino, Russia Federation; Auburn University College of Veterinary Medicine, UNITED STATES

## Abstract

**Purpose:**

House dust mites *Dermatophagoides pteronyssinus* are the main source of major inhalatory allergens inducing inflammatory response. Mite extract contain both allergenic proteins and lipopolysaccharides (LPS). The main allergenic protein, Der p 2, is a functional homolog of sMD-2, a protein providing blood cell response on LPS. Der p 2 may restore the response to LPS in absence of MD-2, but its interaction with LPS in whole blood is unknown. We studied the effect of Der p 2 on LPS-mediated activation of human whole blood cells.

**Methods:**

Interaction of Der p 2 and LPS was studied on eight healthy donors. The whole blood was incubated with extract of house dust mite *Dermatophagoides pteronyssinus* (DP-e), recombinant antigenic protein Der p 2 variant 5 (rDep 2), *Escherichia coli* lipopolysaccharide and their combination. Supernatants were collected for ELISA analysis of protein content. Activation degree was determined by change in concentration of TNF-α, IL-8, IL-1Ra cytokines and sMD-2 protein.

**Results:**

extract of mite *Dermatophagoides pteronyssinus* (DP-e) possessed weak inherent activity and did not cause significant increase of cytokine production. Simultaneous activation of blood cells by LPS and DP-e led to considerable increase of pro-inflammatory cytokine production. We have shown the intrinsic inducing activity of Der p 2 allergen on sMD-2 protein and TNF-α cytokine expression.

**Conclusions:**

Der p 2 allergen enhances the response of human whole blood cells to external LPS by inducing additional expression of LPS-transporting protein sMD-2. The obtained data show an important role of LPS contamination of allegrens in the progress of allergic inflammatory response.

## Introduction

Allergic diseases are the most widespread type of immune disorders in the world. Around 30% of world population demonstrate symptomes of allergy. Social and economic effects of allergy, considering its severeness and distribution, have been drastically growing recently. House dust mites (HDM) are considered as the major source of inhalatory allergens, participating in pathogenesis of anaphylactic reactions in humans and animals [1, 2]. They are present in dust, mattresses, pillows, stuffed animals [3]. The widespread HDM species are *Dermatophagoides pteronyssinus* (Der p) and *Dermatophagoides farinae* (Der f). Inflammatory response caused by allergen action is nowadays connected with adaptive immune response and direct activation of innate immunity cells. Such activation is mediated by both allergens and other components present in dust or in environment [4].

The allergens entering human organism are contaminated with bacterial products, particularly endotoxins (lipopolysaccharides, LPS). LPSs produced by Gram-negative bacteria activate human innate immune system cells via TLR4 receptor, resulting in synthesis of pro-inflammatory cytokines [5]. The most crucial cytokines participating in blood response to allergens and endotoxins are pro-inflammatory TNF-α, chemokine IL-8 and anti-inflammatory IL-1Ra.

The majority of clinically important allergens, including HDM, also cause allergic reactions via TLR4 receptor stimulation [6]. Up to date, there are at least three different known mechanisms for TLR4 activation by allergens: sensitization of LPS-induced TLR4•MD-2 activation, generation of endogenous TLR4•MD-2 ligands or direct induction of TLR4•MD-2 dimerization [7].

Myeloid differentiation protein 2 (MD-2), also known as LY96, is the most important cofactor of TLR4-dependent signal transduction from LPS. This protein is physically bound to the extracellular part of TLR4 [8]. The excessive MD-2 is secreted by cells in a soluble form, sMD-2. It could function in blood serum as a co-stimulating molecule in the response of TLR4-transfected HEK293 cells on LPS [9]. Moreover, *in vitro* studies have shown that sMD-2 can directly bind LPS and the resulting LPS•MD-2 complexes are the active ligands capable of activating TLR4 expressing cells [10–12].

It was found so far, that *Dermatophagoides pteronyssinus* mite allergen group 2 (Der p 2) is an MD-2 mimetic and it could activate synthesis of its mRNA in lymphocytes and monocytes (PBMC) [13, 14]. Due to functional homology, Der p 2 provides LPS-mediated TLR4 activation in absence of MD-2 and facilitates it in presence of MD-2. Beside activation of innate immunity, Der p 2 interacts with B-lymphocytes, involving adaptive immunity into the response [15].

Contamination of allergens entering the organism by endotoxins and the ability of Der p 2 to interact directly with LPS and to regulate sMD-2 mRNA synthesis in peripheral blood mononuclear cells (PBMC) shows the ability of the allergen to regulate progression of inflammatory response to bacterial LPS. Currently, there are no works on studying interaction of endotoxin and Der p 2 allergen in human whole blood.

The purpose of the current work was to study the role of Der p 2 allergen in blood cell activation by endotoxins.

## Materials and methods

### Reagents

The following reagents were used in the work: S-glycoform of *E*. *coli* LPS O55:B5 (Sigma, USA); extract of allergens from *D*. *pteronyssinus* mite (DP-e, 5000 PNU/ml, AO «Biomed», Russia), recombinant protein Der p 2 variant 5 (rDer p 2, MyBioSource, USA), RPMI1640 medium tested for absence of endotoxins, containing 25mM HEPES, NaHCO_3_ and L-glutamine (Sigma, USA), ELISA test systems for determination of cytokines TNF-α, IL-8, IL-1Ra (LLC Cytokine, Saint-Petersburg) and sMD-2 (MyBioSource, USA).

### Activation of whole blood leukocytes for cytokine production

The experiments were conducted on heparinized peripheral blood of 5–10 healthy donors (age 18–30 yo) who gave the written consent to participation in the study after informing on procedures. The research protocol corresponds to the World Medical Association Declaration of Helsinki and was approved by the Local Ethic Committee of the Hospital of Pushchino Scientific Center (№2 of 10.04 2014). The criteria for blood collection for the experiment are concordant to requirements for blood donation: absence of innate diseases; medical certificate with information on all the diseases for half a year; absence of infectious diseases and contact with infected persons for at least two months.

Blood specimens were collected into BD Vacutainer NH (Sodium heparin) 102 I.U. Plus Blood Collection Tubes (Becton, Dickinson and Company, UK) in clinical environment. Heparinized blood was diluted by RPMI 1640 medium at 1:4 ratio and divided into 1 ml samples in 24-well Cellstar plates (Greiner Bio-One, Germany). To study effect of agonists on cytokine synthesis, whole blood samples were supplied with *E*. *coli* LPS (40 ng/ml), DP-e (30 μg/ml), both DP-e and *E*. *coli* LPS in the same concentrations, rDer p 2 variant 5 (final concentration 4 μg/ml or 10 μg/ml). The samples without addition of activating substances, i.e. Der p 2 allergen extract, LPS or their combination, served as a control. Blood samples were incubated for 6 h at 37°C and 5% CO_2_ in CO_2_ incubator (Jouan, France). Then blood cells were centrifuged for 10 min at 1000 rpm (140g) in a centrifuge LMC-3000 (BioSan, Latvia). The supernatants were collected and stored at -20°C till determination of cytokine and sMD-2 content.

Protein content in whole blood samples was measured by using commercially available ELISA kits according to manufacturers' protocols. Optical density was measured by ELISA analyzer STAT FAX 3200 (Awareness Technology, USA) at 450 nm wavelength. Quantitative estimation of the results was provided by calibration curve reflecting the dependence of optical density on concentration of protein to be analyzed.

### Statistical analysis

The results are presented as medians (*Me*) with interquartile range (*IQR*). The reliability of difference between median values was estimated by Mann-Whitney U-test and Wilcoxon Signed Rank Sum Test. A *P*-value *P*<0.05 was considered statistically significant for difference of medians. Correlation coefficient was calculated by the Pearson correlation coefficient (*PCC*). Microsoft office Excel 2010 (AtteStat plugin), STATISTICA 10.1 and SigmaPlot 12.5 program packages were used for statistical analysis and data representation.

## Results

### Simultaneous action of allergen and endotoxin increases cytokine expression

#### TNF-α

**Fig 1:**
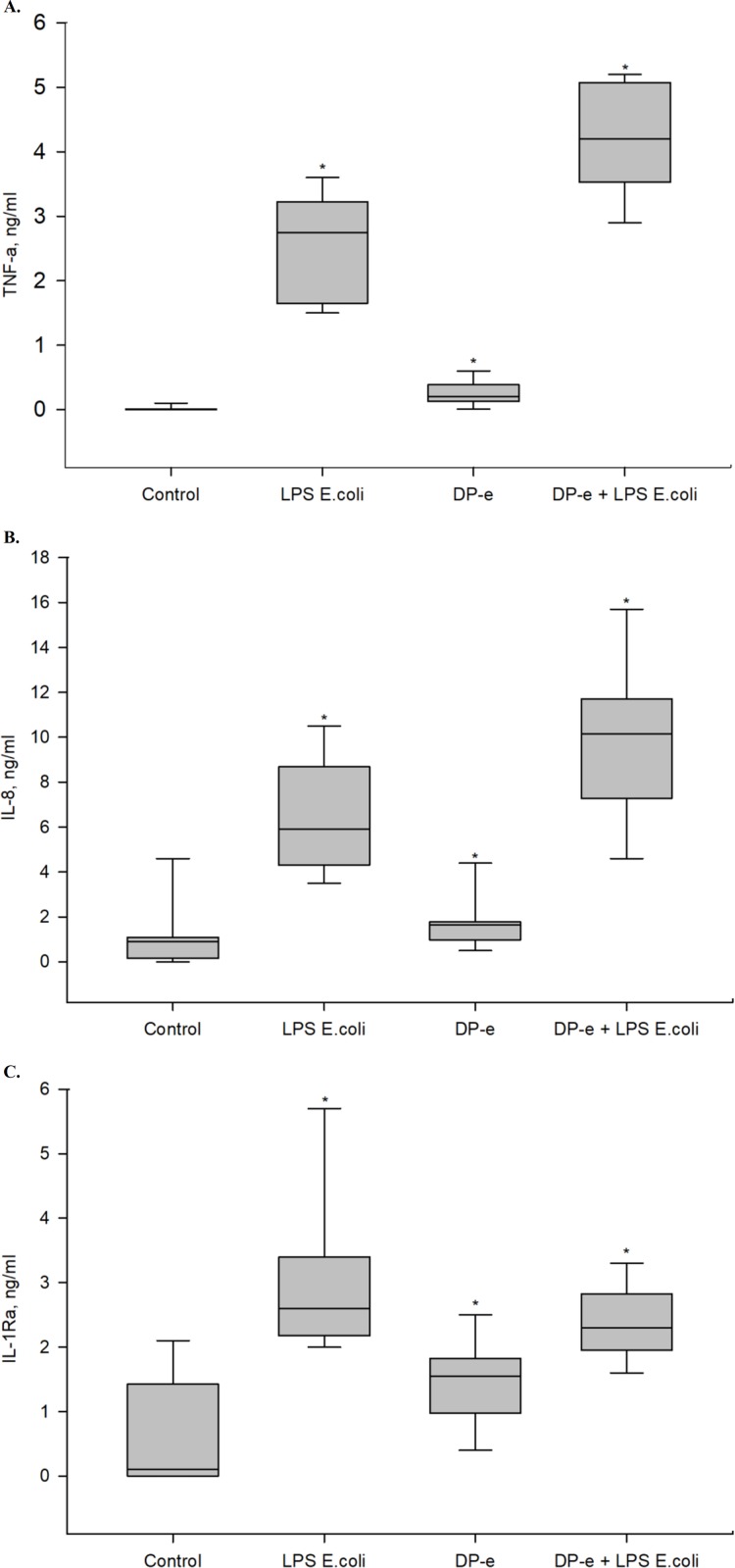
Effects of LPS and Dp-e on cytokines secretion by blood cells. Blood cells treated with endotoxin (LPS *E*. *coli*), DP-e or their combination. TNF-α (A), IL-8 (B) and IL-1Ra (C) were measured from supernatants. The samples without addition of activating substances served as a control. The cytokines expression was evaluated using ELISA. n = 8, *- statistically significant (p<0.05) difference from control, Mann-Whitney U-test.

TNF-α is one of the earliest cytokines released by monocytes in response to endotoxins [16]. This cytokine is absent in blood of healthy donors. Activation of blood cells by LPS from *E*. *coli* induced significant production of TNF-α. Extract of Der p allergen caused slight expression of this cytokine (Fig 1A).

Simultaneous activation of whole blood cells by the allergen and the endotoxin induced notable release of TNF-α by blood cells. Amount of produced TNF-α in response to cooperative action of the agonists was by 77±17% higher than on LPS.

#### IL-8

Background level of IL-8 might be present in blood of healthy donors [17]. In control samples, median concentration of this chemokine was 0.9 ng/ml. Activation of blood cells by agonists (DP-e and LPS from *E*. *coli*) and by their combination induced extra release of IL-8. Patterns of changes in IL-8 synthesis were the same as for changes in TNF-α synthesis: small amount of produced IL-8 during activation by DP-e, significant production of IL-8 under the effect of endotoxin and maximal IL-8 production level under simultaneous action of DP-e and *E*. *coli* LPS (Fig 1B).

Amount of IL-8 produced in response to cooperative action of agonists was by 61±17% higher than in response to LPS. Six times more IL-8 was produced in response to combination of DP-e and LPS than to antigen extract only.

#### IL-1Ra

Anti-inflammatory cytokine IL-1Ra is necessary for lowering inflammation level in cases of overactivation of innate immune cells. IL-1Ra was present in control samples. The largest amount of anti-inflammatory IL-1Ra was detected in blood affected by endotoxin. Simultaneous activation of blood cells by LPS and allergen nduced synthesis of lower IL-1Ra quantities than activation by endotoxin alone (Fig 1C).

To find out correlation between levels of pro-inflammatory and anti-inflammatory cytoknes, TNF-α/IL-1Ra and IL-8/IL-1Ra ratios were measured after blood activation by various agonists and their combination. No significant correlation was found.

#### Correlation between TNF-α and IL-8

**Fig 2:**
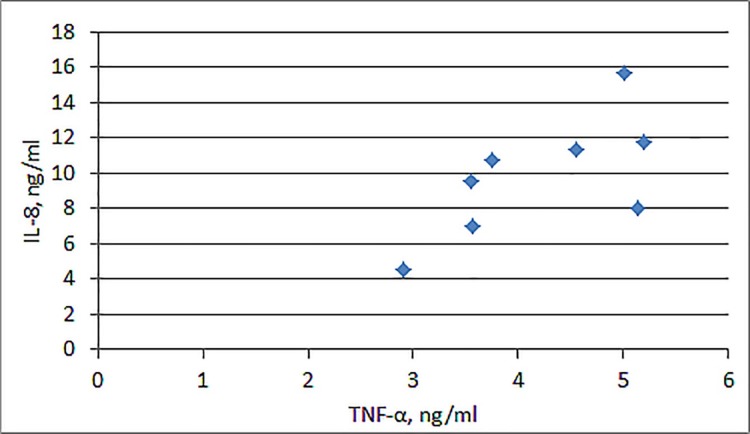
Correlation between TNF-α and IL-8 concentrations by activated blood cells. Blood cells treated with combination of endotoxin and DP-e (LPS *E*. *coli*, 40 ng/ml + DP-e, 30 μg/ml) for 6 hours. TNF-α and IL-8 were measured from supernatants. The cytokines expression was evaluated using ELISA, n = 8.

TNF-α and IL-8 are the crucial prognostic cytokines in progression of bronchial asthma. A study by Jiang et al. showed positive correlation between these cytokines [18]. We have analyzed correlation between levels of these cytokines after blood cells activation by endotoxin, allergen and their combination. Significant correlation between these two cytokines was observed only under simultaneous action of DP-e and LPS (Fig 2, *PCC* = 0.68).

Expression levels of the cytokines after blood activation by separate endotoxin or allergen did not correlate with each other (*PCC*<0.3, S1 Table).

IL-8 to TNF-α ratio, a possible indicator of inflammation progression, was tested. Activation of blood cells by the allergen caused production of minor amount of TNF-α and quite a large amount of IL-8 playing a leading role in allergy development, whereas activation by endotoxin caused notable production of pro-inflammatory cytokine TNF-α, thus decreasing the cytokine ratio. The ratio under simultaneous effect of endotoxin and allergen was close to that during activation by LPS alone and much lower than during activation by allergen. Existence of correlation between IL-8 and TNF-α levels and ratio of these cytokines points on prevalent impact of LPS into development and progression of allergic response caused by allergen from *D*. *pteronyssinus*.

### Role of Der p 2 and sMD-2 in response of blood cells to endotoxins and allergens

Role of sMD-2 in response of blood cells to agonists and their combination was studied to reveal the mechanism of synergistic effect of DP-e and LPS from *E*. *coli* on cytokine synthesis. MD-2 and its soluble form, sMD-2, are the central proteins in activation of blood cells by endotoxins. Der p 2 is a functional homolog of sMD-2 [13]. Moreover, Der p 2 can enhance expression of MD-2 mRNA, affecting the promoter of its gene [14]. These data show that synergistic effect of DP-e and LPS could be realized directly via blood cell activation by Der p 2•LPS via TLR4 receptor or indirectly via increased sMD-2 synthesis.

Increase of sMD-2 concentration to 20 ng/ml was shown to enhance activation of nuclear factor NF-κB, which plays a key role in signal transduction from TLR4 receptor to cell nucleus and activation of synthesis of cytokines, such as TNF-α and IL-8 [19]. Comparison of dependency of TNF-α and IL-8 production on sMD-2 blood level showed absence of correlation between sMD-2 blood level up to 20 ng/ml and synthesis of these cytokines.

**Fig 3:**
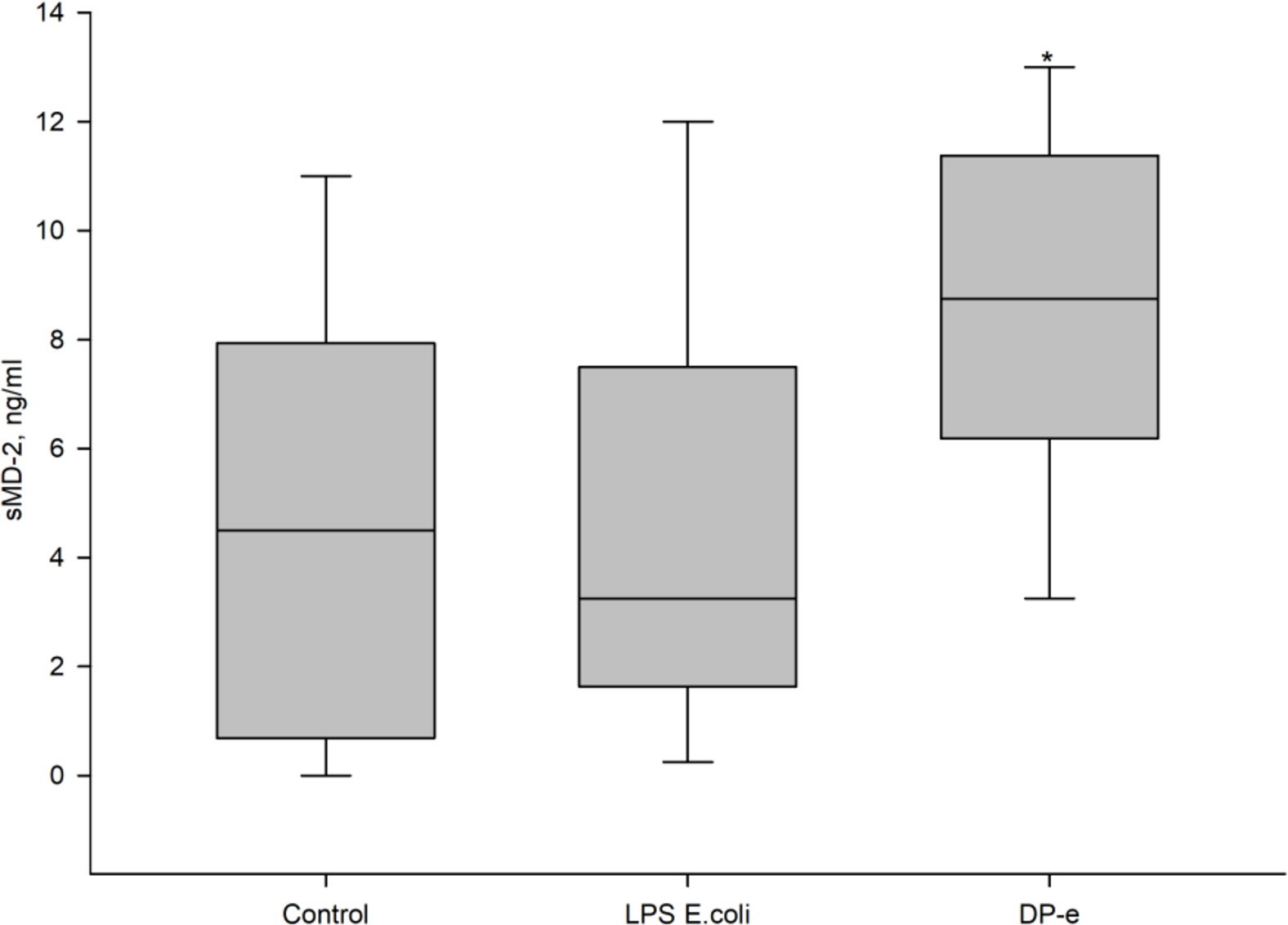
Effect of DP-e on sMD-2 secretion by blood cells. Blood cells treated with endotoxin (LPS *E*.coli), allergen (DP-e) or their combination. sMD-2 was measured from supernatants. The samples without addition of activating substances served as a control. The cytokines expression was evaluated using ELISA. n = 8, *- statistically significant (p<0.05) difference from control, Mann-Whitney U-test.

sMD-2 is present in blood of healthy donors. During activation by endotoxin, sMD-2 concentration slightly decreased to 3.2 ng/ml (not reaching statistically significant values). Treatment of the cells with *D*. *pteronyssinus* extract caused reliable significant increase of sMD-2 concentration to 8.7 ng/ml (Fig 3).

**Fig 4:**
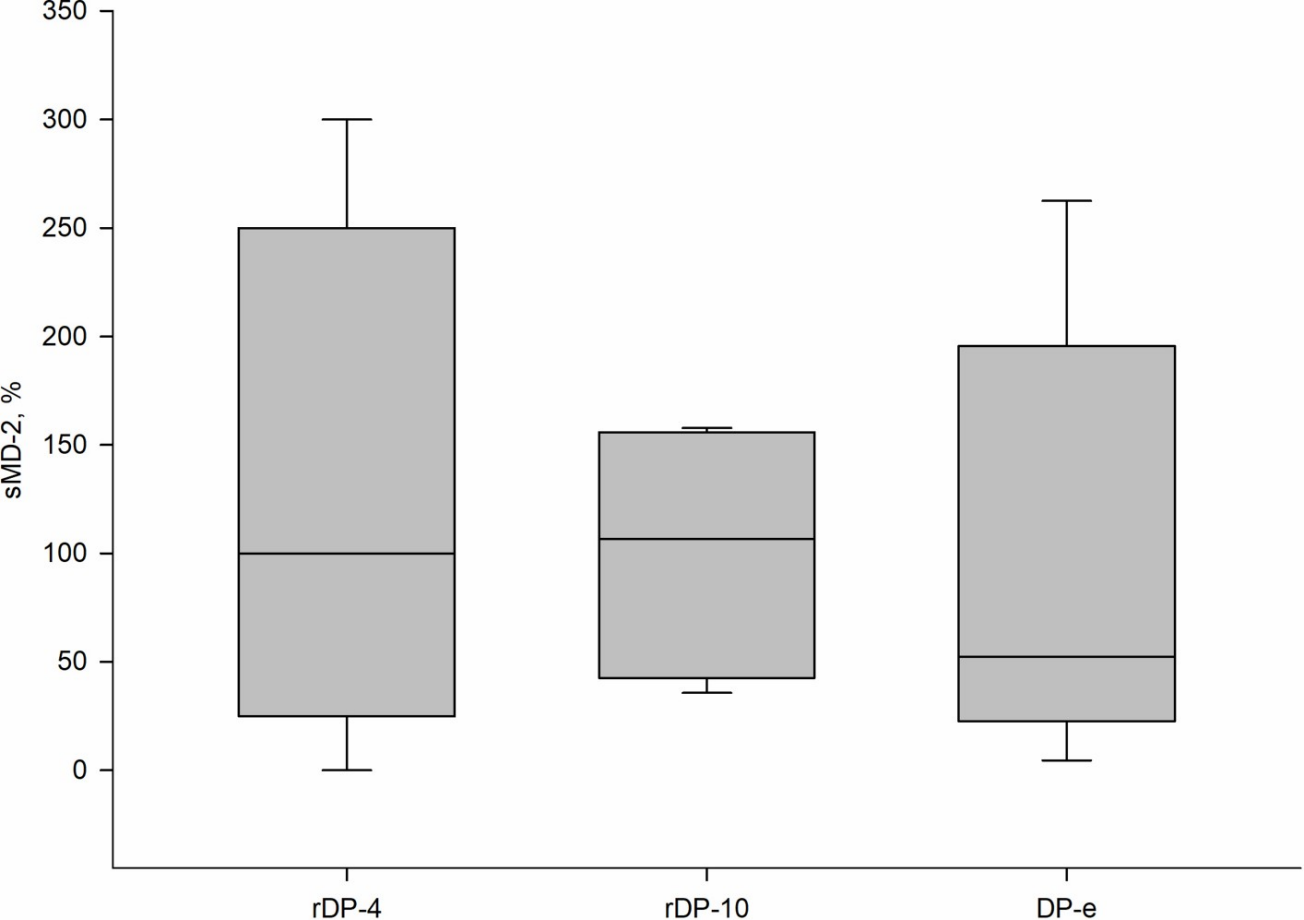
Change of sMD-2 concentration during blood cells activation by allergens. Blood cells treated with rDep 2 (4 μg/ml or 10 μg/ml) and extract of allergen (DP-e, 30 μg/ml). (n = 4–6). Figure shows the percentage, by which sMD-2 was increased compared to control after exposure to various concentration of rDer p 2 or Dp-e.

Beside Der p 2 protein, which is a mimetic of MD-2, a number of other proteins and metal ions capable of TLR4-mediated cell activation are present in the allergen extract [7]. To study the effect of the sole allergenic protein, we conducted experiments on changing TNF-α and sMD-2 levels during activation of blood cells from 4–6 healthy donors by recombinant Der p 2 form. rDep 2 provoked cells to produce TNF-α. As it is shown on Fig 4, the tested concentrations of Der p 2 and DP-e induced sMD-2 production equally.

Growth of sMD-2 level in blood after activation by the allergen was 80% on average. Maximal level of sMD-2 had been achieved at 4 μg/ml of recombinant Der p 2 and further addition of the allergenic protein up to 10 μg/ml did not alter sMD-2 expression. The obtained data show that 4 μg/ml recombinant Der p 2 is a saturating concentration sufficient for maximal induction of sMD-2 synthesis.

## Discussion

Der p 2 is the main allergenic protein of house dust mite *Dermatophagoides pteronyssinus* [20]. Due to global distribution of allergic asthma, the mechanism of progression of allergic response and molecules enhancing this effect draw increased attention. House dust endotoxin is known as one of the most powerful activators of innate immune response [21–22]. We couldn't find any data in the literature on interaction between Der p 2 and LPS in blood. We studied synergistic effect of Der p 2 and endotoxin (LPS) on synthesis of blood protein sMD-2, pro-inflammatory cytokines TNF-α and IL-8, and anti-inflammatory cytokine IL-1Ra in whole blood cells of healthy donors. The use of whole blood eliminates possible artifacts that may be associated with isolation of cells, such as adherence-induced expression of TNF-α.

TNF-α is an important cytokine of innate immune response playing a crucial role in direct protection of organism from external microorganisms before activation of adaptive immune system [23]. It is one of the first cytokines to be expressed in response to bacterial LPS [24], and it is one of nine basic asthma-associated proteins in ethnically diverse populations [25]. Elevated concentration of TNF-α is detected in people with symptomatic and asymptomatic asthma after allergen exposure [26].

Being a chemokine, IL-8 plays a crucial role in the development of allergy. Expression of this cytokine by monocytes is enhanced under the action of LPS [27–29].

IL-1Ra, an antagonist of IL-1 receptor, is an anti-inflammatory cytokine capable of blocking cytokine expression by BEAS-2B cells after exposure to house dust mite extract and of inhibiting LPS-induced inflammation [30–32]. Thus, all the cytokines studied in our work take part in inflammatory response to LPS and Der p 2 allergen.

Studying changes of TNF-α, IL-8 and IL-1Ra production levels in response to endotoxin and allergen, we found that DP-e, unlike endotoxin, has very weak innate activity. During simultaneous effect of LPS and allergen, blood cells displayed the maximal expression of TNF-α and IL-8, whereas IL-1Ra amount was lower than under the action of endotoxin alone. The observed effect could be related to production of IL-1, which takes part in inflammatory response to LPS [33]. IL1-Ra could probably bind to excess IL-1 produced under simultaneous action of DP-e and LPS, which could be the reason of decrease of its content in blood.

Data from the literature showed no correlation between IL-1Ra and IL-8 concentrations in cases of neutrophilic asthma [34]. The results obtained in our work show a slight positive correlation between IL-1Ra and IL-8 under simultaneous action of endotoxin and allergen, which could point onto mixed phenotype of asthma developed under the action of these agonists. The amount of IL-1Ra produced under simultaneous effect of LPS and Der p 2 has a weak correlation with amount of TNF-α produced, which is also proven by data from the literature [31].

TNF-α and IL-8 levels strongly correlate during development of bronchial asthma in patients [18]. Our experiments showed reliable correlation only during simultaneous activation of blood cells from healthy donors by endotoxin and allergen. IL-8 to TNF-α ratio was close to one during activation by LPS only and significantly lower than after DP-e action. The obtained data confirm the important role of the endotoxin in development of bronchial asthma. To obtain the most realistic pattern of allergic inflammation development in model systems, simultaneous activation by allergen and endotoxin must be used.

The obtained data show that the effect of cooperative action of DP-e and endotoxin cannot be reduced to simple addition of effects of each separate agonist; extract of *D*. *pteronyssinus* allergen can enhance the response of blood cells to toxic LPS. Our results confirm the proposal of Liao et al. on synergistic action of allergen and endotoxin [14].

We have shown for the first time that Der p 2 protein present in extract of *D*. *pteronyssinus* mite allergen can provoke additional production of sMD-2 protein in human whole blood. The conducted experiments with recombinant Der p 2 showed that additional synthesis of sMD-2 is definitely caused by Der p 2 and not by other components of mite extract entering the body. The intrinsic activity of Der p 2 can be explained by its functional homology with blood protein MD-2 [35]. MD-2 is a glycoprotein coexpressed with TLR4 on the surface of different cell types, and it is necessary for recognition of endotoxin carried by transporter proteins [36]. The excessively synthesized MD-2 is secreted into blood in soluble form (sMD-2) [19], providing LPS transfer to effector cells.

Der p 2 can facilitate signal transduction to TLR4 via direct interaction with TLR4 complex [13]. Due to functional homology, Der p 2 restores LPS-dependent activation of TLR4 endotoxins in absence of MD-2 and facilitates it in presence of MD-2.

Our results show that concentration of circulating sMD-2 in blood is sufficient for activation of blood cells by endotoxin. The level of pro-inflammatory cytokines production by blood cells does not depend on sMD-2 content in concentration range up to 20 ng/ml. Correlation between sMD-2 expression and blood cell response to endotoxin was shown in the literature [19], but we did not find such a correlation. This could be explained by the fact, that, on one hand, the main producing cells in the body are epithelial cells and inflammatory cells of liver and lungs. This protein could be produced *de novo* in isolated blood by B-lymphocytes and monocytes, but in far lower amounts [8]. On the other hand, sMD-2 does not only participate in TLR4-dependent activation of blood cells by bacterial LPS. An important function of this protein is its opsonizing activity, providing activation of neutrophils and neutralization of Gram-negative bacteria [37, 38]. The sMD-2 protein produced in response to Der p 2 could enhance both cellular response to endotoxins and opsonization of Gram-negative bacteria.

The observed decrease of sMD-2 amount in blood during activation by endotoxin can be explained by endotoxin binding by monomeric form of the protein, whereas the main part of polymeric sMD-2 protein stays circulating in blood. The active form enhancing the response of the cells to the endotoxin is monomeric sMD-2 [39], which is produced by *de novo* stimuli. Polymeric form is the form prevailing in circulating blood in normal conditions.

The obtained results confirm that activation of blood cells by allergen causes reliable increase of sMD-2 level in blood. The data from the literature show that Der p 2 can enhance sMD-2 mRNA expression in PBMC. Our results showed that maximal level of additionally synthesized sMD-2 is reached already at 4 μg/ml rDep 2. Thus, the allergen does not only activate transcriptional activity of sMD-2 genes, but it also induces the release of the protein already present in the cells.

We suppose that, despite the functional homology, Der p 2 and sMD-2 proteins do not compete for endotoxin. This proposal is supported by higher affinity of LPS to sMD-2 than to Der p 2 [35]. The enhancement of pro-inflammatory response to combined action of endotoxin and allergen could be related to indirect effect of Der p 2 on sMD-2 synthesis. sMD-2 was shown in the literature to cause enhanced TLR4 expression on epithelial cell surface [40]. When blood cells are activated by Der p 2 antigen, TLR4 expression on leukocyte surface is not increased (unpublished data). Apparently, the additionally produced sMD-2 allows more complete transport of endotoxin to TLR4, providing increased inflammatory response.

Our experiments showed that Der p 2 and LPS can act synergistically, causing the enhanced expression of pro-inflammatory cytokines. Probably the main mechanism of such synergy can be the ability of the allergen to enhance sMD-2 protein, providing additional activation of blood cells by endotoxins. Such a synergy gives evidence on an important role of LPS in development of bronchial asthma.

## Supporting information

S1 Table Effects of LPS and allergen on secretion of cytokines and sMD-2 by blood cells. Blood cells treated with endotoxin (LPS E.coli), allergen (DP-e) or their combination. TNF-α (A), IL-8 (B), IL-1Ra (C) and sMD-2 (D) were measured from supernatants. The cytokines expression was evaluated using ELISA. These results are represented in Figs 1–3.
(DOCX)

S2 Table Effect of allergens on sMD-2 secretion by blood cells. Blood cells treated with recombinant der p 2 protein in concentration of 4 μg/ml (A) or 10 μg/ml (B) and allergen DP-e (C). sMD-2 was measured from supernatants. The sMD-2 expression was evaluated using ELISA. n = 4–6. These results are represented in Fig 4.
(DOCX)
