# Supplementary material for: Synergistic effect of Dermatophagoides pteronyssinus allergen and Escherichia coli lipopolysaccharide on human blood cells
Source: PLoS One. 2018 Nov 9;13(11):e0207311. doi: 10.1371/journal.pone.0207311 (PMC6226202; doi:10.1371/journal.pone.0207311)
Supplement: S1 Table — Blood cells treated with endotoxin (LPS E.coli), allergen (DP-e) or their combination. TNF-α (A), IL-8 (B), IL-1Ra (C) and sMD-2 (D) were measured from supernatants. The cytokines expression was evaluated using ELISA. These results are represented in Figs 1–3. (DOCX) [file pone.0207311.s001.docx]

S1 Table. Effects of LPS and DP-e on secretion of cytokines and sMD-2 by blood cells.

A

| Donors (D) | Concentration of TNF-a. ng/ml | | | |
| --- | --- | --- | --- | --- |
|  | Control | LPS *E.coli* | DP-e | DP-e + LPS *E.coli* |
| D1 | 0 | 1.6 | 0.35 | 3.6 |
| D2 | 0.01 | 3.6 | 0.4 | 3.5 |
| D3 | 0 | 3 | 0.2 | 5.1 |
| D4 | 0 | 3.3 | 0.2 | 5.2 |
| D5 | 0 | 2.8 | 0.2 | 5 |
| D6 | 0 | 1.8 | 0.1 | 4.6 |
| D7 | 0 | 1.5 | 0.01 | 2.9 |
| D8 | 0.1 | 2.7 | 0.6 | 3.8 |
|  |  |  |  |  |
| B |  |  |  |  |
| Donors (D) | Concentration of IL-8. ng/ml | | | |
|  | Control | LPS *E.coli* | DP-e | DP-e + LPS *E.coli* |
| D1 | 0.6 | 4.1 | 1.7 | 7 |
| D2 | 1.1 | 10.5 | 1.6 | 9.6 |
| D3 | 0 | 3.5 | 0.5 | 8.1 |
| D4 | 0 | 6 | 1.2 | 11.8 |
| D5 | 4.6 | 8.8 | 4.4 | 15.7 |
| D6 | 1.1 | 8.3 | 1.8 | 11.4 |
| D7 | 1 | 4.9 | 0.9 | 4.6 |
| D8 | 0.8 | 5.8 | 1.7 | 10.7 |
| C |  |  |  |  |
|  |  |  |  |  |
| Donors (D) | Concentration of IL-1Ra. ng/ml | | | |
|  | Control | LPS *E.coli* | DP-e | DP-e + LPS *E.coli* |
| D1 | 1.2 | 2.7 | 1.6 | 2.2 |
| D2 | 1.5 | 2.1 | 1.6 | 1.9 |
| D3 | 0 | 2.5 | 0.8 | 2.1 |
| D4 | 0 | 5.7 | 1.9 | 3.3 |
| D5 | 0 | 3.6 | 1.5 | 2.9 |
| D6 | 0.2 | 2.4 | 1.5 | 2.4 |
| D7 | 0 | 2 | 0.4 | 1.6 |
| D8 | 2.1 | 2.8 | 2.5 | 2.6 |
|  |  |  |  |  |

| D |  |  |  |  |
| --- | --- | --- | --- | --- |
| Donors (D) | Concentration ofsMD-2. ng/ml | | |  |
|  | Control | LPS *E.coli* | DP-e |  |
| D1 | 0 | 3.5 | 6 |  |
| D2 | 2 | 1.5 | 7.25 |  |
| D3 | 5.25 | 2 | 6.75 |  |
| D4 | 7.75 | 6.75 | 11 |  |
| D5 | 3.75 | 3 | 10.25 |  |
| D6 | 8 | 7.75 | 13 |  |
| D7 | 11 | 12 | 11.5 |  |
| D8 | 0.25 | 0.25 | 3.25 |  |
