# Supplementary material for: Synergistic effect of Dermatophagoides pteronyssinus allergen and Escherichia coli lipopolysaccharide on human blood cells
Source: PLoS One. 2018 Nov 9;13(11):e0207311. doi: 10.1371/journal.pone.0207311 (PMC6226202; doi:10.1371/journal.pone.0207311)
Supplement: S2 Table — Blood cells treated with recombinant der p 2 protein in concentration of 4 μg/ml (A) or 10 μg/ml (B) and allergen DP-e (C). sMD-2 was measured from supernatants. The sMD-2 expression was evaluated using ELISA. n = 4–6. These results are represented in Fig 4. (DOCX) [file pone.0207311.s002.docx]

S2 Table. Effect of DP-e and rDer p 2 on sMD-2 secretion by blood cells.

A

| Donors (D) | Concentration of sMD-2. ng/ml | |
| --- | --- | --- |
|  | Control | rDP 4 |
| D1 | 1.5 | 1.5 |
| D2 | 1 | 2 |
| D3 | 1.5 | 3 |
| D4 | 0 | 3 |
| B |  |  |
| Donors (D) | Concentration of sMD-2. ng/ml | |
|  | Control | rDP 10 |
| D1 | 0.38 | 0.62 |
| D2 | 0.42 | 0.57 |
| D3 | 0.19 | 0.49 |
| D4 | 0.2 | 0.5 |
|  |  |  |
| C |  |  |
| Donors (D) | Concentration of sMD-2. ng/ml | |
|  | Control | DP-e |
| D1 | 2.0 | 7.3 |
| D2 | 5.3 | 6.8 |
| D3 | 7.8 | 11.0 |
| D4 | 3.8 | 10.3 |
| D5 | 8.0 | 13.0 |
| D6 | 11.0 | 11.5 |
